# Associated factors of dietary diversity among schoolchildren in Plateau Central region of Burkina Faso: a cross-sectional study

**DOI:** 10.1186/s40795-024-00896-0

**Published:** 2024-06-25

**Authors:** Daniel Somwaoga Ouedraogo, Ella W. R. Compaore, Ousmane Ouedraogo, Mamoudou H. Dicko

**Affiliations:** 1 Department of Biochemistry/Microbiology, Laboratory of Biochemistry, Biotechnology, Food Technology and Nutrition (LABIOTAN), Joseph KI-ZERBO University, Ouagadougou, Burkina Faso; 2 03 BP 7021 Ouagadougou 03, Burkina Faso

**Keywords:** Dietary diversity score, Primary school students, Associated factors, Burkina Faso

## Abstract

**Context:**

School-age is a dynamic period of growth and development, leading to good health and a productive adult life. Adequate dietary intake provides essential nutrients for growth, health and cognition. However, the practices of adequate nutrition is still not a matter of course for schoolchildren in many countries. The aim of this study was to identify associated factors of dietary diversity among students in public primary school in the Central Plateau Region.

**Method:**

Multi-stage sampling was used to select schoolchildren. A semi-structured questionnaire was used to collect information’s of food consumption at home and at school using a 24-h dietary recall method. Binary logistic regression was used to identify variables associated with students' dietary diversity scores (DDS) with statistical significance at *p* < *0.05*, after performing Chi-square test of independence to identify candidates variables at *p* < *0.25*.

**Results:**

The study involved 560 pupils aged 6 to 14 older, including 52.9% girls and 47.9% boys. Dietary diversity was divided into three classes: low (DDS ≤ 4), medium (DDS = 5) and high (DDS ≥ 6). Thus, 13.4% of students have a low DDS and average in 48.9%, versus 37.7% high. Students in Ganzourgou were twice as likely to have a low DDS (AOR = 2.01, 95% CI:1.00–4.04) compared to those in Oubritenga. Household drinking water source, pupil status and father's occupation were significantly associated with pupils' dietary intake.

**Conclusion:**

Primary schoolchildren don’t have good dietary practices in the Plateau Central Region. Promoting dietary diversification in households and balanced meals in school canteens would be necessary to improve the DDS of schoolchildren.

**Trial registration:**

Clinical Trial Number: 2022_33_/MS/MESRSI/CERS of 02/14/2022.

**Supplementary Information:**

The online version contains supplementary material available at 10.1186/s40795-024-00896-0.

## Background

The Dietary Diversity Score (DDS) is a simple tool for quantifying dietary diversity and reflecting micronutrient adequacy at population level. It was developed by the Food and Agriculture Organization of the United Nations (FAO) and can be used with a single 24-h dietary recall by counting the number of food groups rather than foods consumed [15]. It is a measure of food security, nutritional information, early warning system and intervention target at global or national level [24].

Infants and young children are the most affected by physical and mental deficiencies due to deficiency malnutrition, one of the main causes of which is low dietary diversity [39]. Most of the time, monotonous staple diets lack essential micronutrients, leading to macro and micronutrient deficiencies, particularly in the most vulnerable group [42].

These deficits are carried over into the school years, where they delay cognitive function, education and future productivity.

Consequently, health promotion targeting schoolchildren and adolescents is seen as an effective social vaccine against current health problems and the health challenges of the next generation [14, 41].

Optimal nutrition is essential during adolescence. In actual fact, 50% of adult weight, skeletal mass and 20% of adult height are acquired during this period. However, 45–60% of adolescent girls have sub-optimal dietary intakes, leading to the development of various micronutrient deficiencies (vitamin A, iron and iodine deficiencies) [13, 43].

Adolescence is a period of rapid physical, emotional, social, sexual and psychological development and maturation, which in turn increases the need for energy, protein, vitamins and minerals. These characteristics make adolescents vulnerable to a whole range of nutritional problems [9, 43].

"School" can serve as a platform for targeted interventions, such as school feeding programs, to help meet children's nutritional needs outside the first 1,000 days of life. School feeding helps eliminate hunger for millions of children worldwide, and contributes to their education, nutrition, health and future productivity as adults [21]. Indeed, school canteens can limit the adverse effects of emergencies on health, nutrition and education, thereby reducing barriers to accessing and completing education, especially for girls [16].

However, in-depth reviews of empirical research [16] and program evaluation reports [12, 21] have shown that, although the school feeding program had positives effects on educational participation, its effects on nutritional outcomes were rather unclear and that the substitution effect of school feeding for home consumption was partly responsible for the lack of effect [25, 27].

In Burkina Faso, very few studies of feeding practices take school-age children into account. Data on food consumption by school-age children can be used to draw-up nutritionals recommendations for the government, with a view to taking action to improve food consumption practices for all populations, and schoolchildren in particular.

As a consequence, this study aimed to assess the factors associated with food consumption and dietary diversity among public elementary school pupils in the Central Plateau Region of Burkina Faso.

## Material and methods

### Study area and population

This descriptive cross-sectional study was conducted in the Plateau Central Region, whose capital Ziniaré is located 35 km from Ouagadougou, the political capital of Burkina Faso. The Plateau Central Region is composed of the provinces of Ganzourgou, Kourwéogo and Oubritenga. This Region has a Sudano-Sahelian climate in the northern part (Kourwéogo and Oubritenga provinces), marked by a long dry season (October to May) and a short rainy season (June to September). Rainfall is irregular and insufficient, with an annual average varying between 600 and 800 mm. Agriculture is a major source of livelihood in the region, marked by a high proportion of cereal production [19]. The study population consisted of public primary schoolchildren whose canteen was operational at the time of the study. In 2019, the region's population was 978,614 (53.14% female), 90.42% of whom lived in rural areas. This population included 300,744 school-age children (aged of 6–14 years) [18]. In 2022, according to the statistical yearbook of the Ministry of National Education, the region had 735 public schools in the 2021/2022 school year.

### Inclusion criteria

These criteria were at two levels: school level and student level.

- ❖ At school level: according to the list of schools in the region provided by the Regional Department of Education, for the 2021-2022 school year, the following criteria were used to select schools:

#### School inclusion criteria

- be a public school with a complete primary cycle;
- have a functional canteen at the time of collection;
- have the approval of the school director.

The list of schools has been processed to take only those schools that meet these criteria into account. The distribution of number of schools per province to be included was made according to the proportion of the province's student population to the number of students in the region. A reasoned choice was made to include two schools in the provincial capital.

- ❖ Schoolchildren criteria: in each school selected for the study, only the following criteria guided the choice of participants:
  - ▪ Inclusion criteria for pupils:
    - attend the school selected by lottery;
    - consent to participate to the study
    - have parental consent;
    - be among those selected by lottery;
    - not be ill on the day of the collection.

### Sampling

#### Sample size

In the lack of data on dietary diversity among students in the region, the sample size is estimated using the prevalence of anemia among school-age children as a reference, which was 53.3% in the Central Plateau region according to the results of the National Iodine and Anemia Survey in Burkina [29]. Data on the population of schoolchildren were provided by the Regional Department of Preschool, Primary and Non-Formal Education (DREPPNF) of the Central Plateau. The sample size was estimated using OpenEpi (version 3), a free sample calculation software. Thus, on the basis of the data provided, a representative sample requires at least 370 children to obtain a confidence level of 95% with a margin of error of 5%. Taking into account a non-response rate of 10%, the final sample size planned was 407 subjects (370 + (370 × 10 / 100). The survey involved 20 to 25 pupils from 24 public primary schools. The final sample size was therefore 560 pupils. Data collection reached 560 schoolchildren aged 6 to 14 years from 24 public primary schools.

Two methods were used to select the schools to be included in the study: the reasoned choice method and the random selection method. The reasoned choice method involved dividing the schools into urban (provincial capital) and rural (village) zones. The random selection method was based on the random selection of two schools in each urban area (three zones) and the selection of schools in rural areas, in proportion to the number of schools meeting the criteria in each province.

### Study variables

Socio-demographic variables, such as age, gender, province, place of residence, father's and mother's level of education, father's and mother's occupation, family size whether or not school meals were consumed, deworming during the last 6 months, breakfast consumption, composition of school meals and student status were recorded as explanatory variables.

The student dietary diversity score (DDS) is the dependent variable in this study. It is used as a proxy measure of the nutritional quality of the individual diet. It was calculated using the 24-h dietary recall method, using an individual questionnaire developed by the Food and Agriculture Organization of the United Nations (FAO) [27]. This questionnaire was completed with the help of mother or guardian who accompanied the student on the day of the survey. Food groups were defined in accordance with FAO guidelines for measuring dietary diversity at household and individual level **(**Table 1) [23]. Consumption of foods from each food group was sufficient to be counted, unless a food was used solely as a condiment. Foods consumed by each individual the day before interview were recorded in a food group table. Finally, the dietary diversity score was constructed by counting the food groups consumed by the students over the previous 24 h. All foods and beverages consumed were divided into nine food groups in accordance with FAO guidelines to measure individual dietary diversity: (1) starchy staple foods (including cereals, roots and tubers); (2) dark green leafy vegetables; (3) vitamin A-rich fruits and vegetables; (4) other fruits and vegetables; (5) organ meats; (6) meat and fish; (7) eggs; (8) legumes, nuts and seeds; (9) milk and dairy products. Students' food consumption (at school and at home) was assessed on the basis of food groups defined using a questionnaire designed by the FAO (Annex). One point was assigned to each food group consumed, with 9 corresponding to the maximum score over the previous 24 h. All individual dietary diversity scores were categorized as either high Food Diversity (FD) (≥ 5 food groups) or low FD (< = 4 food groups) from the 9 food groups [23].

**Table 1:** Distribution of food products by food group used to calculate the dietary diversity score

Questionnaire food groups	Foods	Dietary diversity score elements
1. Cereals	Sorghum, sorghum cream, sorghum couscous, sorghum toast; millet (small millet), millet cream/degummed/, millet couscous, millet toast (based on flour/bread), maize (broken kernels or flour), sweet roasted maize, maize toast, fonio, fonio toast, rice, pasta (macaroni, spaghetti, etc.), wheat, bread, millet/wheat "pâté", flour (wheat), millet/rice cakes, maize/rice/millet porridge, millet/maize/rice fritters, millet/maize/rice flour enrichment, millet/maize flour enrichment), wheat, bread, millet/wheat, flour (wheat), millet/rice patties, corn/millet/rice porridge, millet/corn/rice fritters, enriched flour (also to be included in pulses)	1. Starches
2. White tubers, roots and plantain	White sweet potato, potato, cassava, taro, plantain banana (aloco), yams, roots, water lily root, turnip root, tô with white sweet potato	
3. Vegetables and tubers rich in vitamin A	Carrot, red bell pepper, orange-fleshed sweet potato, orange- or dark-yellow-fleshed squash (orange-fleshed squash tô), orange-fleshed sweet potato tô	2. Fruits and vegetables rich in provitamin A
4. Fruits rich in provitamin A	Mango, papaya, orange fleshy melon, nɛrɛ / nɛrɛ powder	
5. Dark green leafy vegetables	Baobab leaves, sorrel leaves, dark green shallot leaves, onion leaves, squash leaves, bean leaves, koumba leaves, potato leaves, spinach, dark green salad, lego leaves, bulvaka leaves, cassava leaves, kapok, all dark green wild leaves	3. Dark green leafy vegetables
6. Other vegetables	Fresh tomatoes, fresh or dried okra, eggplants, local eggplants (jaxatus or goyo), zucchini, light-fleshed squash, cucumbers, cabbage, onions, shallots, green peppers, green beans, beets, kapokie flowers, lettuce (light green leaf lettuce), peas, dah pulp, gougoune fruit	4. Other vegetables
7. Other fruits	Watermelon, orange, lemon, wild "dates" (dates, jujube, pineapple, apple, banana, guava, avocado, wild fruit (grape, monkey bread/baobab fruit), shea pulp, liana fruit, roasted fruit flesh, cashew fruit, fruit, cinnamon apple, orange, tamarind, monkey bread Fresh fruit juice (pressed fruit), plum fruit juice, grape gel …	
8. Offal	Liver, kidneys, heart, lungs, or any other offal (veal, sheep, goat, camel, poultry), viscera (viscera soup), clotted blood	5. Meat, Offal, Fish and seafood
9. Meat	Beef, sheep, goat, rabbit, bush meat, chicken, guinea fowl, camel, birds, bustard, duck, monitor, turtle, insects, caterpillars/worms, maroons, wild rats, agoutis, squirrels, partridges, snakes, mice, warthogs, deer, etc	
10. Insects	Grasshoppers, mayflies, caterpillars, etc	
11. Fish and seafood	Fresh fish, smoked, salted or dried fish (except for a pinch of powder), canned fish (sardines, tuna, etc.), all shellfish and seafood (shrimp, squid, octopus, lobster, etc.), dried or smoked fish powder (in large quantities)	
12. Eggs	Eggs from chicken, guinea fowl, varan, duck, etc	6. Eggs
13. Pulses	Beans (cowpea), fari, peanuts (paste or plain), sesame, potato/woandzou peas, sweet peas, date (in large quantities for sauce), soumbala (in large quantities for sauce), cashew nuts, boscia nuts, wild walnuts, chickpeas, lentils, water lily seed, other pulses, etc	7. Pulses
14. Red palm products	Red palm oil, red palm nuts	8. Nuts and seeds
15. Nuts and seeds	Peanuts (paste or other), soybeans, sesame, cashew nuts, shea nuts, wild nuts, cottonseeds, etc	
16. Milk and dairy products	Fresh milk, powdered milk, condensed milk (sweetened or not), natural curd, yoghurt, cheese	9. Milk and dairy products

## Data collection, processing and analysis

### Tools, procedure and data collection period

Questionnaires for this study were entered into KoboToolbox and then deployed on Kobo Collect for mobile data collection. Data was collected by twelve collectors who were recruited according to their skills and trained over two days on the interview approach. Data collection took place from April 25 to 30, 2022 in the selected schools. The questionnaire used for the interviews concerned the student's food consumption practices over the 24 h preceding the day of the survey. Information on school meals was collected from school canteen staff, while information on family meals and other consumption was collected from pupils in the presence of the mother or the person who had prepared the meal on the previous day.

Although school-age children were the main subjects, mothers and caregivers responded for children, as children are likely to give inaccurate descriptions of their dietary intake. The mother or carer of each pupil was first asked to name all foods, including drinks and snacks, consumed at home and away from home by the indexed child in the 24 h (from waking to waking) prior to the survey. She was then asked about foods likely to have been forgotten, and asked to provide a detailed description of the foods and drinks consumed, including the ingredients of mixed dishes. To ensure that food consumed outside the home was taken into account, children were asked to help their mothers or guardians name all the foods they had eaten. For the school canteen meal, the composition was given by the canteen maid who prepared the meal the day before the survey. The Dietary Diversity Score (DDS) ranges from zero (0) to eight (9). It was calculated for each student over the previous 24 h by summing the number of food groups consumed to categorize children's dietary diversity as low (≤ 4 food groups), medium (= 5 food groups) and high (≥ 6 food groups) based on nine food groups.

### Data quality

To guarantee data quality, the tools were pre-tested by the data collectors following two days' training. Data collection was carried out under the supervision of two supervisory agents, and regular checks on data completeness and consistency were carried out on a daily basis. Before analysis, the data were checked to ensure that there were no missing values.

### Data processing and analysis

The data collected were exported to SPSS version 25 software for statistical analysis. Descriptive statistics were used to describe students' socio-demographic and economic characteristics. The Chi-square test of independence was performed to identify candidate variables for binary logistic regression. Thus, variables with a *p* < 0.20 were entered into the binary logistic regression model. The results of the binary logistic regression were reported using Odds Ratios and 95% confidence intervals (CIs). A *p*-value of less than 0.05 was considered statistically significant. Results were presented in tables.

## Results

### Socio-demographic characteristics

A total of 560 students aged 6 to 14 were included in the study, with a 100% response rate. The mean age was 10.79 (± 2.09 SD) years, of which 296 (52.9%) were girls. The socio-demographic characteristics of the population at study inclusion are summarized in the Table 2 Around 89% of students lived with at least one biological parent, and 68.8% had been dewormed during the six years preceding the study (Table 2).

**Table 2:** Socio-demographic characteristics of households

**Variable**	**Modality**	**Frequency (n)**	**Rate (%)**
Sex	Female	296	52.9
	Male	264	47.1
Province	Ganzourgou	234	41.8
	Kourwéogo	141	25.2
	Oubritenga	185	33
Age group	6 - 11 years	327	58.4
	12 - 14 years	233	41.6
Household size	<= 6	144	25.7
	7 -15	288	51.4
	16 - 30	112	20
	31+	16	2.9
Dewormed within last 6 months	Yes	175	31.3
	No	385	68.8
Student status	Living with parents	501	89.5
	Children in care	59	10.5

### Socio-economic status and dietary practices

In this study, 88.6% of the children lived in rural areas. More than a majority of the mothers of the children interviewed (54.1%) had no schooling, compared with 40.4% of the men. As for the parents' level of education, only 12.1% of fathers and 10.5% of mothers had reached secondary level. Consequently, 75.9% of student mothers are housewives and 79.3% of fathers are farmers. 5.5% of students say they didn't eat school meals because their parents forbid it (Table 3).

**Table 3:** Socio-economic status and dietary practices

**Variable**	**Modality**	**Frequency (n)**	**Rate (%)**
Place of residence	Urban	64	11.4
	Rural	496	88.6
Mother's education level	No schooling	303	54.1
	Elementary	189	33.8
	Secondary and higher	68	12.1
Father's education level	No schooling	226	40.4
	Elementary	275	49.1
	Secondary and higher	59	10.5
Mother's occupation	Shopkeeper	102	18.2
	Housekeeper	425	75.9
	Civil servant	19	3.4
	Tailor	14	2.5
Father's occupation	Shopkeeper	60	10.7
	Farmer	444	79.3
	Informal sector	27	4.8
	Civil servant	29	5.2
Eat the canteen meal	Yes	529	94.5
	No	31	5.5
Breakfast	Yes	195	34.8
	No	365	65.2
Canteen food diversification	Low	439	78.4
	Acceptable	121	21.6
Food diversification level	Low (≤ 4)	75	13.4
	Medium (= 5)	274	48.9
	High (≥ 6)	211	37.7
Level of diversification of family meal	Low (≤ 4)	347	62
	Medium (= 5)	130	23.2
	High (≥ 6)	83	14.8
Level of dietary diversification of the school canteen mea	Low (≤ 4)	503	89.8
	Medium (= 5)	26	4.6
	High (≥ 6)	31	5.5
Source of drinking water	Others	85	15.2
	Faucet	48	8.6
	Drilling	427	76.3

Diversity score of the school canteen meal and that of the meal consumed at home the day before the survey were calculated independently. The threshold for each student's Diversity Score was defined using the tertile method. Results showed that 13.4% of students were classified in the lowest dietary diversity score (DDS ≤ 4 food groups) and 48.9% in the medium dietary diversity score (DDS = 5 food groups) while 37.7% classified in the highest dietary diversity score (DDS ≥ 6 food groups).

Calculating the DDS by considering only food consumption at home, we found that 62% of students had a low DDS. Using the same calculation, 23.2% had an average DDS and 14.8% a high DDS. Using the same calculation for the school canteen meal only, 89.8% of pupils had a low DDS (Table 3).

### Prevalence of dietary diversity among school-age children

**Fig. 1:**
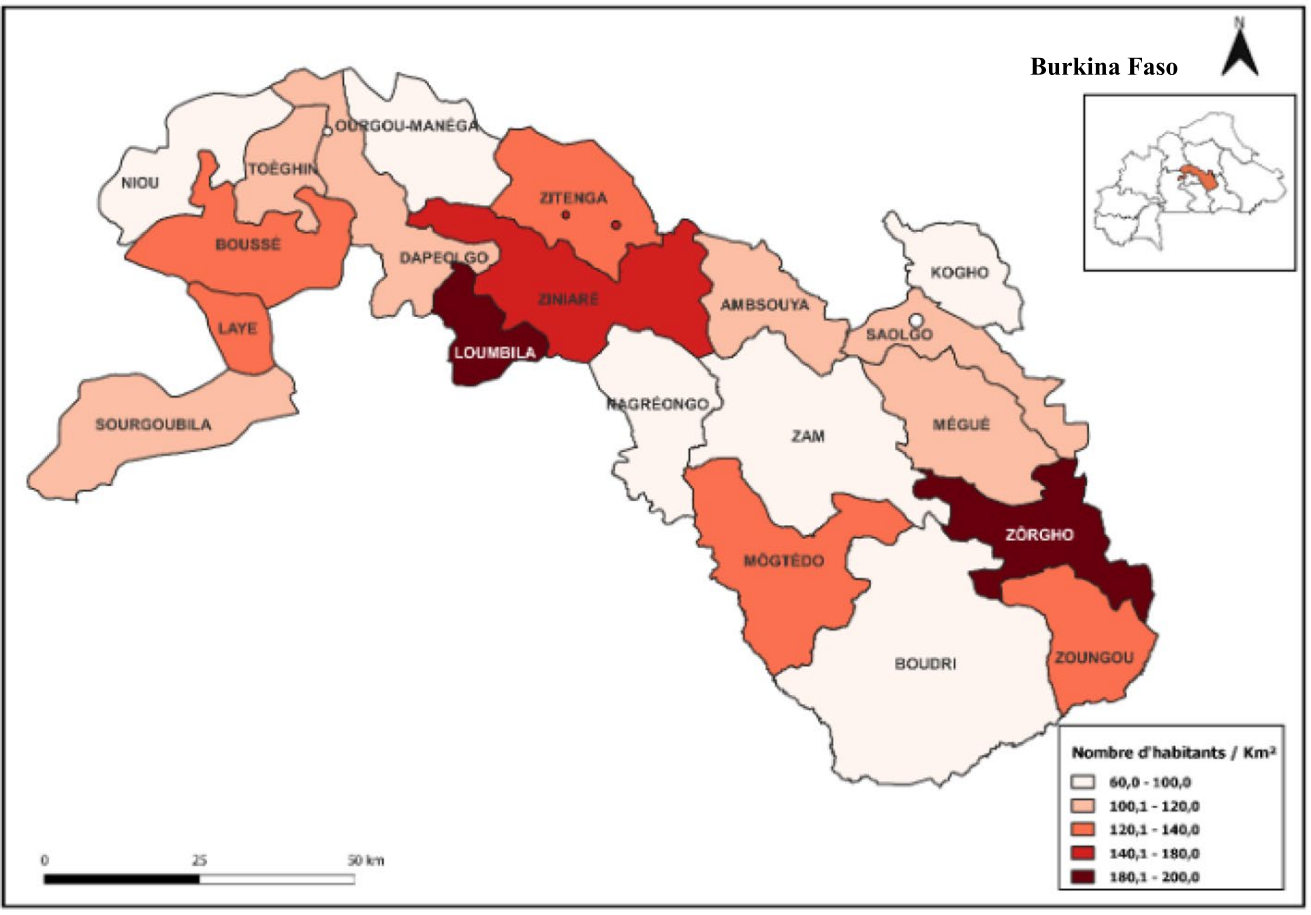
Map of study area

**Fig. 2:**
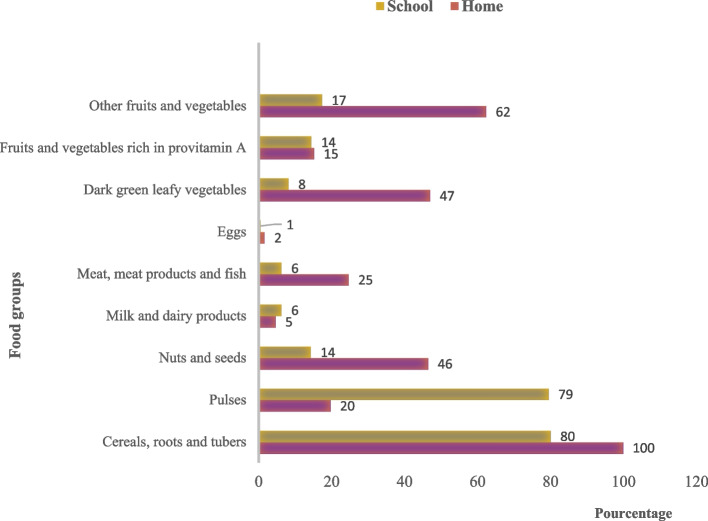
Distribution of students according to consumption of the 9 food groups for the food score at home and at school over 24 h

Almost all students (99.82%) had eaten cereal-based foods at home (Figs. 1 and 2). Also, 80% of students said they had eaten cereal-based foods at school. Also, 80% of students reported eating cereal-based foods at school. As for legumes, over three-quarters of students (79.46%) said they had eaten them at school, while only 19.64% had eaten them with their families. As for the nuts and seeds group, almost half of students (46.43%) consumed them with their families, while only 14.29% of students had access to this food group at school. Around 1% of students reported consuming eggs, and around 5% of students reported consuming milk and dairy products, either at school or at home. As for meat, meat products or fish, around 25% of students reported have eaten them at home, while only 6.24% of students had eaten them at school.

Fruits and vegetables rich provitamin A have been consumed at home and school by about 5% students. The vegetables to dark green leaves are little consumed by students (8.21%) to school against 46.96% in family. Other fruits and vegetables were consumed by more than half the students (62.32%) at home, compared with 17.32% at school. In the figure (Fig. 1), the y-axis represents the different food groups and the x-axis the percentage of students who ate meals from these groups.

### Explanatory variables of dietary diversity

The Independence test with Chi-square at a significance level of less than 0.25 (Chi-square < 2) shows that place of residence, province of residence, mother's profession, profession of head of household, parents' education level, household drinking water source, composition of canteen meal foodstuffs, pupil status, diversification score level of family meal and school canteen meal were candidate variables for multivariate logistic regression (Table 4).

**Table 4:** Explanatory variables of dietary diversity score

**Variable**	**Modality**	**Dietary Diversity**		**Total**	***p-Value***
		**Low**	**High**		
Sex	Female Male	186 (62.8%) 163 (61.7%)	110 (37.2%) 101 (38.3%)	296 (52.9%) 264 (47.1%)	0.789
Place of residence	Urban Rural	37 (57.8%) 312 (62.9%)	27 (42.2%) 184 (37.1%)	64 (11.4%) 496 (88.6%)	0.429
Province	Ganzourgou Kourwéogo Oubritenga	140 (59.8%) 86 (61%) 123 (66.5%)	94 (40.2%) 55 (39%) 62 (33.5%)	234 (41.8%) 141 (25.2%) 185 (33%)	0.351
Household size class	6 7-15 16-30 ≥ 31	93 (64.6%) 182 (63.2%) 64 (57.1%) 10 (62.5%)	51 (35.4%) 106 (36.8%) 48 (42.9%) 6 (37.5%)	144 (25.7%) 288 (51.4%) 112 (20.0%) 16 (2.9%)	0.640
Eat the canteen meal	No Yes	26 (83.9%) 323 (61.1%)	5 (16.1%) 206 (38.9%)	31 (5.5%) 529 (94.5%)	0.011*
Household toilet	No Yes	143 (58.6%) 206 (65.2%)	101 (41.4%) 110 (34.8%)	244 (43.6%) 316 (56.4%)	0.111*
Household drinking water sources	Others Faucet Drilling	59 (69.4%) 23 (47.9%) 267 (62.5%)	26 (30.6%) 25 (52.1%) 160 (37.5%)	85 (15.2%) 48 (8.6%) 427(76.3%)	0.048*
Dewormed within last 6 months	No Yes	229 (59.5%) 120 (68.6%)	156 (40.5%) 55 (31.4%)	385 (68.8%) 175 (31.3%)	0.040*
Breakfast	No Yes	229 (62.7%) 120 (61.5%)	136 (37.3%) 75 (38.5%)	365 (65.2%) 195 (34.8%)	0.780
Mother's occupation	Shopkeeper Housekeeper Civil servant Tailor	68 (66.7%) 262 (61.6%) 12 (63.2%) 7 (50%)	34 (33.3%) 163 (38.4%) 7 (36.8%) 7 (50%)	102 (18.2%) 425 (75.9%) 19 (3.4%) 14 (2.5%)	0.612
Mother's education level	No schooling Elementary Secondary and higher	186 (61.4%) 119 (63.0) 44 (64.7%)	117 (38.6%) 70 (37.0%) 24 (35.3%)	303 (54.1%) 189 (33.8%) 68 (12.1%)	0.856
Father's occupation	Shopkeeper Farmer Informal sector Civil servant	37 (61.7%) 276 (62.2%) 16 (59.3%) 20 (69.0%)	23 (38.3%) 168 (37.8%) 11 (40.7%) 9 (31.0%)	60 (10.7%) 444 (79.3%) 27 (4.8%) 29 (5.2%)	0.881
Father's educational level	No schooling Elementary Secondary and higher	144 (63.7%) 166 (60.4%) 39 (66.1%)	82 (36.3%) 109 (39.6%) 20 (33.9%)	226 (40.4%) 275 (49.1%) 59 (10.5%)	0.608
Age group	5 - 11 years 12 - 14 years	207 (63.3%) 142 (60.9%)	120 (36.7%) 91 (39.1%)	327 (58.4%) 233 (41.6%)	0.570
Food composition level	Low Acceptable	274 (62.4%) 75 (62.0%)	165 (37.6%) 46 (38.0%)	439 (78.4%) 121 (21.6%)	0.931
Student status	Living with parents Children in care	310 (61.9%) 39 (66.1%)	191 (38.1%) 20 (33.9%)	501 (89.5%) 59 (10.5%)	0.526
Dietary diversity score class	≤ 4 = 5 ≥ 6	56 (100%) 265 (100%) 28 (11.7%)	0 (0%) 0 (0%) 211 (88.3%)	56 (10%) 265 (47.3%) 239 (42.7%)	0.000*
Family meal diversity class	≤ 4 = 5 ≥ 6	290 (83.6%) 34 (26.2%) 25 (30.1%)	57 (16.4%) 96 (73.8%) 58 (69.9%)	347 (62.0%) 130 (23.2%) 83 (14.8%)	0.000*
Diversity class for school canteen meals	≤ 4 = 5 ≥ 6	339 (67.4%) 2 (7.7%) 8 (25.8%)	164 (32.6%) 24 (92.3%) 23 (74.2%)	503 (89.8%) 26 (4.6%) 31 (5.5%)	0.000*
**Total**		**349 (62.3%)**	**211 (37.7%)**	**560 (100%)**	

*Value of ***p < 0.25***: Variables candidate for logistic regression

The mean (± SD) intake of DDS in public primary school children was 4.27 (± 1.47). The results showed that 62.3% of school children have low dietary diversity scores (DDS ≤ 4) versus 37.7% who have high dietary diversity scores (DDS ≥ 5).

### Factors associated with dietary diversity

The Chi-square test were performed and variables with a *p*-value < 0.25 were entered for multivariate logistic regression. All variables selected during multivariate logistic regression remained significant at a *p*-value < 0.05 according to multivariate modeling, and these variables are listed in Table 5..

**Table 5:** Multivariate analysis of associated factors of students' dietary diversity score

**Variable**	**Modality**	**Frequency (%)**	**OR (IC95%)**	***p- Value***
Province	Ganzourgou Kourwéogo Oubritenga	234 (41,8) 141 (25,2) 185 (33,0)	2,01 (1.00-4.04) 1.72 (0.82-3.59) 1	0.04* 0.14
Mother's occupation	Shopkeeper Housekeeper Civil servant Tailor	102 (18,2) 425 (75,9) 19 (3,4) 14 (2,5)	0.45 (0.09-2.31) 0.57 (0.11-2.92) 0.66 (0.08-5.45) 1	0.34 0.50 0.70
Father's occupation	Shopkeeper Farmer Informal sector Civil servant	60 (10,7) 444 (79,3) 27 (4,8) 29 (5,2)	3.10 (0.57-16.91) 3.69 (0.68-20.09) 2.59 (0.37-18.16) 1	0.18 0.02* 0.33
Mother's education level	No schooling Elementary Secondary and higher	303 (54,1) 189 (33,8) 68 (12,1)	1.02 (0.34-3.06) 1.11 (0.38-3.29) 1	0.97 0.83
Household drinking water sources	Others Faucet Drilling	85 (15,2) 48 (8,6) 427 (76,3)	1.06 (0.49-2.26) 2.15 (0.78-5.89) 1	0.87 0.16
Father's educational level	No schooling Elementary Secondary and higher	226 (40,4) 275 (49,1) 59 (10,5)	0.85 (0.25-2.79) 1.05 (0.33-3.36) 1	0.79 0.92
Dewormed within last 6 months	No Yes	385 (38,8) 175 (31,3)	1.31 (0.72-2.40) 1	**0.37**
Eat the canteen meal	No Yes	31 (5,5) 529 (94,5)	0.09 (0.02-0.31) 1	0.000***
Household size class	≤ 6 7 - 15 16-30 ≥ 31	114 (20,4) 288 (51,4) 112 (20,0) 16 (2,9)	1.13 (0.19-6.62) 1.53 (0.27-8.47) 1.05 (0.18-6.03) 1	**0.88** **0.62** **0.95**
Dietary diversity score class	≤ 4 = 5 ≥ 6	347 (62,0) 130 (23,2) 83 (14,8)	0.02 (0.01-0.05) 0.82 (0.39-1.72) 1	0.000*** **0.606**
Diversity class for school canteen meals	≤ 4 = 5 ≥ 6	503 (89,8) 26 (4,6) 31 (5,5)	0.07 (0.02-0.23) 8.06 (1.24-52.30) 1	0.000*** 0.029*

*Significant value at ***p*** **< 0.05**; ** Significant value at ***p *< 0.01**; *** Significant value at ***p *< 0.001**

The results of the binary logistic regression indicated that occupation of the household head, source of household drinking water, province of residence, canteen meal consumption, and student diversity class at home and at school were significantly associated with students' dietary diversity intake. Thus, students whose dietary diversity at home was considered low (DDS < 4), according to the tertile classification, had an 80% risk of having a low overall DDS (AOR = 0.02, 95% CI: 0.01–0.05) compared to those whose DDS of the school canteen meal was low (AOR = 0.07, 95% CI: 0.02–0.23) with a risk of 30%. Similarly, pupils living in the Ganzourgou province had twice the risk of having a low DDS (AOR = 2.01, 95% CI: 1.00–4.04) (Table 5.) compared to pupils in the Kourwéogo province.

## Discussion

### Limitations of the study

Student dietary diversity estimated on the basis of a single 24-h dietary recall may not reflect daily and seasonal dietary variation. Although the questionnaire is the one recommended by the FAO [15], potential memory biases cannot be ignored. This study did not take into account household food security, income level, parents' age, dietary and nutritional practices. Lastly, the cross-sectional nature of this study makes it impossible to determine the temporality of eating habits and nutritional outcomes.

It should be noted, however, that there are differences in the methodology used to calculate the dietary diversity score in school-age children. In some studies, the DDS is determined on the basis of 10 food groups using a 24-h dietary recall dataset with points awarded only when food subcategories are ingested [41]. In this study, DDS was assessed on the basis of 9 food groups a global calculation method with no minimum intake for each food subcategory. Consequently, we would expect it to return relatively higher dietary diversity score values compared to threshold-based calculation methods. There is currently no single threshold for setting DDS calculation thresholds, which makes direct comparisons between studies more or less problematic.

### Dietary diversity score

The present study investigated factors associated with dietary diversity in public elementary school pupils aged 6–14 years in the Central Plateau region of Burkina Faso using regionally representative data.

The results show that the food groups most frequently consumed by students were cereal-based foods, legumes, nuts and seeds, other fruits and other vegetables. This predominance of these food groups in consumption was reported in similar studies [7, 31, 38]. On contrast, animal products such as meat, eggs and vitamin-rich plant products, dairy products and other fruit were consumed less, as in other studies [1, 7]. Students should be encouraged to eat foods rich in protein and vitamins, which are optimal for the body's growth and development [14].

The results also revealed low dietary diversity among 37.7% (95% CI: 33.7–41.8) of public primary school children in the Central Plateau region. The seasonal nature of certain foods and fruits could explain the low dietary diversity score observed in this study.

This proportion of students with a high dietary diversity score was better than the 22.2% reported by Ayogu among primary school children in rural Nigeria [7]. Other similar studies of pupils in elementary school also revealed low levels of dietary diversity, in south-western Nigeria (24.2%) [38], Kenya (28.5%) [31], Côte d'Ivoire (30.9%) [37] and Ghana (32.9%) [3], compared with the results of the present study. These differences can be explained by the number of days observed to calculate the dietary diversity score. Indeed, in Nigeria and Kenya, two 24-h periods were used and the average DDS was calculated. Also, the classification thresholds were different from those used in the present study. In Côte d'Ivoire, the high DDS threshold was at least 7 out of 12 food groups, compared with 4 food groups in Ghana.

The lack of dietary diversity among students in the Central Plateau region of Burkina Faso could be explained by the fact that their diet is largely cereal-based, with little or no animal products and few fresh fruits and vegetables.

Results of the present study were comparable to those of other studies in elementary school in Ethiopia in the Gedeo zone with 37% [5] and in the town of Dilla [8], with a proportion of 38.7%.

The dietary diversity score reported in this study was lower than that found in other similar studies. Mwaniki and Makokha reported a score of 45.2% among elementary school children in Nairobi, Kenya [5], which was higher than the results of our study. However, Mwaniki and Makokha (Mwaniki & Makokha) considered the dietary diversity score to be high in children who consumed at least four food groups. The results were lower than those of other similar studies carried out in Africa: 54% in public elementary school in Port Hacourt and 87.5% in private schools in Nigeria [22] and also 68.7% and 41.7% in Ethiopia in the Jimma [1, 35] zones. The results were also lower than those of a similar study carried out in Croatia, where the DDS was 51.8% [17]. This performance could be explained by the high availability of diversified foodstuffs over a longer period in these countries. Also in these countries, school meals may be more diversified than in Burkina Faso.

The overall mean dietary diversity score was 4.27 (SD ± 1.47) and ranged from 2 to 8 food groups. Although this score was higher than the score reported in a similar study in Port Harcourt Metropolis, Nigeria, where it was 3.67 ± 0.71 SD in public schools [38], it was still lower than the scores observed in Ethiopia (4.69 ± 1.46) among adolescent girls [42]. Other studies revealed even higher mean dietary diversity scores, such as in China among school-age children, with scores of 6.11 ± 1.7 SD [28] and 5.6 ± 1.5 [45] respectively. This could be explained by the fact that in the schools these studies were carried out in China, groups such as all starches, oils and fats, other vegetables, meat, poultry and fish were the most frequently consumed foods.

In this study, the majority of pupils who benefit school canteen meals with a dietary diversity score were from the pilot school for the President of Faso's initiative to "Provide every school-age child with at least one meal a day of good quality and in sufficient quantity". It aims to make school canteens nutritionally sensitive by offering every school-age child at least one balanced meal a day. This initiative was launched in June 2021 in the said school located in the province of Oubritenga in June 2021.

It is imperative to extend school feeding programs to reach all vulnerable schoolchildren, not just those extremely affected, and prevent undernutrition in a country where food insecurity is gaining ground due to the difficult security context.

Despite the limitations of this study, the results do highlight a number of findings concerning school-age children and school canteens in the region in particular, and in Burkina Faso in general.

We therefore suggest that greater attention be paid to the nutritional status of school-age children and to school canteens. Until now, data on nutritional status and health have focused on adolescent girls over 15 and children under 5, as they are more vulnerable and more exposed to disease and death, not to mention the economic losses associated with poor nutritional status [12, 21, 30, 33].

Children under the age of five were often at the heart of strategies and actions to combat malnutrition [40]. Despite children's increased chances of survival after the age of five, school-age children have increased nutritional needs to support the growth thrust of adolescence, requiring diets rich in energy and micronutrients and sufficient in quantity and quality [10, 26].

The results also high light the need to make school canteens more nutritionally sensitive.

In particular, it is important to study the factors that hinder the smooth running of the canteen, in order to find solutions adapted to the realities of each region.

Addressing the underlying factors that contribute to the health and nutritional status of school-age children would help ensure optimal growth and development after the age of five [9, 33].

Therefore, the government needs to focus on raising awareness of good dietary practices at the community level. The Ministry of Education must diversify the foodstuffs used in school canteens to include nutrient-rich foods such as animal sources, fruit and vegetables, especially through endogenous canteens. Monitoring the nutrition and health status of school-age children is crucial to breaking the vicious circle of malnutrition.

### Factors associated with dietary diversity

In this cross-sectional study, a significant association was found between the dietary diversity score and the profession of the head of household (*p*= 0.02). Indeed, children whose parents were traders were six times more likely to have an undiversified diet than children whose fathers were civil servants. The risk is also noted among children of farmers, who are five times more exposed than children of civil servants. This could be explained by parents' lack of knowledge about good feeding practices. This leads to poor dietary diversification through low consumption of fruit and vegetables, and eggs, which are seen as luxury foods. These results were in line with other studies showing low dietary diversification among students whose fathers are farmers or traders in low-income countries in Asia and Africa [2].

Province of residence was an associated factor with dietary diversity. Students in Ganzourgou province were more exposed to low levels of dietary diversity than those in Oubritenga province. One possible reason for this discrepancy was the low availability of food groups. However, the study suggests that the interpretation of DDS survey results in the three provinces may not be the same at different times of the school year.

This study also shows that eating the school canteen meal has a positive impact on pupils' Food Diversity Score. In fact, not eating the school canteen meal was statistically associated (*p*= 0.000) with low dietary diversity among school children. This corroborates the results of previous studies in Burkina Faso [32] although not on dietary diversity. In fact, the school canteen meal plays a vital role in the diet of pupils in rural areas. If functional, the canteen is a reliable source of daily meals for pupils on school days [21, 25, 36, 44]. This has been proven in studies carried out in England [34] and in USA [11].

The level of dietary diversification at home (*p* = 0.000) has a positive influence on dietary diversity. In fact, the results show that only 5.5% of students would have a high dietary diversity score if they ate only the canteen meals, compared with 14.8% for those who ate only the family meal.

However, the diversity of school canteen meals had a positive impact on students' dietary diversity score (*p* = 0.000). Our results confirm the importance of school meals in reducing food insecurity, as the experience of hunger at school was strongly associated with thinness and stunted growth.

Our results do not corroborate those of previous studies describing positive associations between DDS and household size [1]**.** One possible reason for the lack of association between dietary diversity and household size could be that the study didn’t take into account the level of household income or food security. Although household size has an impact on the level of household income, especially in rural areas, this doesn’t prevent it from having an opposite effect if the household is made up of more children and elderly people. Thus, a negative impact will be felt on the qualitative and quantitative food consumption of its members.

This study did not reveal an association between dietary diversity score and place of residence, unlike other studies  [42] which showed that those living in urban areas had greater dietary diversity than those living in rural areas. The reason could be that the number of students in urban areas was low compared to those in rural areas to have an impact on the result.

This study shows that there was a relationship between the parents' level of education and the dietary diversity score. This result corroborates those of other similar studies, which attribute the low level of dietary diversity score to parental ignorance [2, 4, 6, 20], given that a low level of parental education has an impact on knowledge of good dietary practices.

## Conclusion

School-age children and children under the age of 5 are an important target for breaking the intergenerational cycle of malnutrition. Achieving the Sustainable Development Goals (SDGs) in the field of nutrition could be compromised if this primary school target is not taken into account. The results showed that a high percentage of school-age children aged 5 to 14 did not achieve the minimum dietary diversity recommended by the World Health Organization. The study also showed that one in 10 schoolchildren did not get more than three food groups in their canteen meal. The results also clearly indicated that household drinking water source, province of residence, student status, and student diversity class at home and at school were significantly associated with students' dietary diversity practices. A broader study to assess micronutrient deficiency using biomarkers in schoolchildren is recommended for a better appreciation of the nutritional status of this group.

### Supplementary Information

Supplementary Material 1.

## Data Availability

The data is available to anyone who expresses a need by contacting me.
