# Supplementary material for: Associated factors of dietary diversity among schoolchildren in Plateau Central region of Burkina Faso: a cross-sectional study
Source: BMC Nutr. 2024 Jun 25;10:91. doi: 10.1186/s40795-024-00896-0 (PMC11197321; doi:10.1186/s40795-024-00896-0)
Supplement: Supplementary file 1 — Supplementary Material 1. [file 40795_2024_896_MOESM1_ESM.pdf]

Food consumption questionnaire

Province

- ◯ Ganzourgou
- ◯ Kourwéogo
- ◯ Oubritenga

Name and surname(s) of student

____

Pupil's date of birth

yyyy-mm-dd
____

____

Age

____

Sex

- ◯ Male
- ◯ Female

What does your father or uncle do?

- ◯ Civil servant
- ◯ Trader
- ◯ Farmer
- ◯ Farmer
- ◯ Butcher
- ◯ Gold panner
- ◯ Restautant owner
- ◯ Builder
- ◯ Tailor
- ◯ Other
- ◯ I don't know

What's your mother's or tutor's job?

- Civil servant
- Trader
- Farmer
- Farmer
- Butcher
- Gold panner
- Restaurant owner
- Builder
- Tailor
- Other
- I don't know

Who do you currently live with?

- Father and mother
- Father only
- Mother only
- uncle/aunt
- Brother/Sister
- grandparents
- tutors
- Other

How many people (adults and children) live in your household?

____

Are you eating the canteen meal?

- Yes
- No

If no, why not?

- My parents didn't pay
- My parents won't let me eat the meals in the canteen
- I don't like dishes prepared in the canteen
- Others

How was the canteen meal yesterday?

- ○ Very good
- ○ Good
- ○ A bit good
- ○ Not good
- ○ Don't know

How do you like the canteen meals you eat?

- ○ Very good
- ○ Good
- ○ A bit good
- ○ Not good
- ○ Don't know

Is the meal served in your dish enough for you every day?

- ○ Yes
- ○ No
- ○ Sometimes

Do you usually have any food left on your plate after you've eaten?

- ○ Yes
- ○ No
- ○ Sometimes
- ○ Never

If so, what's the reason?

- ○ I'm over-served
- ○ The meal is not good
- ○ I'm not hungry

Where do you eat your school lunches?

- ○ Under the trees
- ○ Dining halls
- ○ In the classroom
- ○ Under a hangar
- ○ Along the way

Which canteen meals do you dislike?

____

Why don't you like these meals?

Before the canteen started, what did you do at lunchtime to get something to eat?

- ◯ Distribute to the house at lunchtime
- ◯ Bring money for lunch
- ◯ I bring to eat
- ◯ I stay at school without eating
- ◯ Others

If you come home at noon to get something to eat, what do you do after you've eaten?

- ◯ Reviewing my lessons
- ◯ Having fun
- ◯ Going back to school
- ◯ Fetching water
- ◯ Taking care of animals
- ◯ Watching television
- ◯ Nothing
- ◯ Others

If other, please specify

If you eat lunch at the canteen at midday, what do you do with it?

- ◯ I eat my school lunch
- ◯ I'm going back home with my meal

What do you do after eating lunch at school?

- ◯ Returning home at midday
- ◯ Staying at school
- ◯ Joining mother or father at work

If you stay at school after lunch, what do you do after you've eaten?

- ◯ Reviewing my lessons
- ◯ Sleeping
- ◯ Having fun

What do you do when you get home from the canteen at midday?

- ◯ Reviewing my lessons
- ◯ Sleeping
- ◯ Having fun
- ◯ Fetching water
- ◯ Taking care of animals
- ◯ Watching television
- ◯ Others

If other, please specify

Did you come to school yesterday with pocket money?

- ◯ Yes
- ◯ No

If so, what did you pay?

When do you wash your hands at school?

- ☐ After bowel movements (shitting)
- ☐ After relieving yourself (urinating)
- ☐ After having eaten
- ☐ After having fun
- ☐ Before eating
- ☐ After touching animals
- ☐ Before handling food
- ☐ Others

What do you wash your hands with at school?

- ◯ Plain water without soap
- ◯ Water and soap
- ◯ Water and center

Did you have lunch at school yesterday?

- ◯ Yes
- ◯ No

Did you eat this meal yesterday?

- Yes
- No

Which person was in charge of the student's meals at home yesterday?

- Mother
- Grandmother
- Another adult member of the family
- One family member under 15 years of age
- Non-family members (neighbors, friends)

Was there some illness that prevented you from eating as usual yesterday?

- Yes
- No

Did you celebrate yesterday (baptism, wedding), die or go to the market?

- Yes
- No

Did you eat anything unusual yesterday compared with other days of the week (celebrations, ceremonies, or unusual lack of meals)?

- Yes
- No

Cereal consumption at school yesterday

Sorghum, sorghum cream, sorghum couscous, sorghum tô; millet (small millet), millet/degue/thiamguiri cream, millet couscous, millet plate, millet plate (flour/breakage), maize (broken or flour), sweet grilled corn, cornmeal, fonio, fonio plate, rice, pasta (macaroni, etc.), wheat, bread, millet/wheat pâte, wheat flour, millet/rice patties, corn/millet/rice porridge, millet/corn/rice fritters, fortified flour

- Yes
- No

Consumption of tubers and roots at school yesterday

White sweet potato, potato, cassava, taro, plantain (aloco), yams, ronin roots, water lily root, turnip root, white sweet potato plate

- Yes
- No

Eating legumes at school yesterday

Beans (cowpeas), ground peas/woandzou, sweet peas, chickpeas, lentils, soybeans, others

- Yes
- No

Eating nuts and seeds at school yesterday

Water lily seed, peanut (in paste or in nature, cakes), sesame, wild nuts, boscia nut, cashew nut, wild nut, date or soumbala, bikalga (fermented sorrel seed)

- Yes
- No

Milk and milk products at school yesterday

Fresh milk, milk powder, condensed milk (sweetened or not), natural curd, yogurt, cheeses...

- Yes
- No

Consumption of offal at school yesterday

Liver, kidneys, heart, lungs, or any other offal (veal, sheep, goat, poultry), viscera (viscera soup)...

- Yes
- No

Meat and meat products consumption at school yesterday

Beef, sheep, goat, rabbit, chicken, guinea fowl, birds, duck bush meat (gazelle, monitor fish, turtle, wild rats, agoutis, squirrels, partridge, snake, mouse, deer, etc.), frog, crapeau...

- Yes
- No

Fish and seafood consumption at school yesterday

Fresh fish, smoked fish, salted fish, canned fish (sardines, tuna...), shrimps, snails, crab...

- Yes
- No

Eating eggs at school yesterday

Chicken, guinea fowl, duck, quail, goose eggs...

- Yes
- No

Consumption of dark green leafy vegetables at school yesterday

Baobab leaves, dark green shallot leaves, onion leaves, koumba leaves, spinach, dark green salad, lele leaves, fakoye leaves, all dark green wild leaves

- Yes
- No

Consumption of vitamin A-rich vegetables, roots and tubers at school yesterday

Carrot, red bell pepper, orange-fleshed sweet potato, orange or dark yellow-fleshed squash (orange-fleshed squash tod), orange-fleshed sweet potato tod, potato leaves, cassava leaves, sorrel leaves (dah), squash leaves, bean leaves, moringa leaves.

- Yes
- No

Consumption of vitamin A-rich fruit at school yesterday

Mango, papaya, pumpkin, orange melon, néré/poudre de néré

- ◯ Yes
- ◯ No

Consommation d'autres légumes à l'école hier

Fresh tomatoes, fresh or dried okra, eggplants, local eggplants (jaxatus or yalo), zucchinis, cucumbers, cabbages, onions, fresh shallots, green peppers, green beans, lettuce (salad), spinach...

- ◯ Yes
- ◯ No

Consumption of other fruits at school yesterday

Banana, guava, watermelon, orange, lemon, dates, jujube, wild fruits ("grape"/bembe, monkey bread/baobab fruit), wild dates (Aduwa), shea pulp, liana fruit, rind fruit flesh, pineapple, avocados...) Fresh fruit juice (pressed fruit)...

- ◯ Yes
- ◯ No

Consumption of red palm oil at school yesterday

Red palm oil, food prepared with red palm nut or red palm nut pulp...

- ◯ Yes
- ◯ No

Insect consumption at school yesterday

Insects, caterpillars, insect eggs, locusts, ephemera (yiou)...

- ◯ Yes
- ◯ No

Oil and fat consumption at school yesterday

Vegetable oil (peanut, sesame, coconut, etc.), shea butter/oil, cow's butter (sirimè), vegetable fats/margarine, mayonnaise, animal fats. - for sauces, seasonings, frying...), shea butter/oil, cow's butter (sirimè), vegetable fats/margarine, mayonnaise, animal fats...

- ◯ Yes
- ◯ No

Sugar consumption and sweet products at school yesterday

Sugar powder or pieces (in tea, coffee, porridge...), sugary drinks (soft drink/ candy, bissap, ginger juice, juice of leaves or fruits of sweet tamarind, lemongrass), honey, jam, sweets, sweet donuts, sweet cakes, sweet biscuits, tea, coffee, chikoré, kinkeliba, unsweetened tamarind leaf or fruit juice

- ◯ Yes
- ◯ No

Consumption of condiments and spices at school yesterday

Chilli, pepper, vinegar, garlic, spice sachet, salt, Maggi cube, white Maggi, bay leaf, concentrated tomato, sorrel-based condiment (seed/ datou, leaves or pulp), onion-based condiment or dried/processed onion leaves or soy-based condiment, small amount of fish powder, okra powder, dried baobab leaf, bell pepper, soumbala.

- ◯ Yes
- ◯ No

When do you wash your hands at the school?

- ☐ After bowel movements (shitting)
- ☐ After relieving yourself (urinating)
- ☐ After having eaten
- ☐ After having fun
- ☐ Before eating
- ☐ After touching animals
- ☐ Before handling food
- ☐ Others

What do you use to wash your hands at home?

- ○ Plain water without soap
- ○ Water and soap
- ○ Water and the center

Sources of drinking water currently used by your household

- ☐ Faucet (in dwelling, yard/concession)
- ☐ Fountain / Public fountain
- ☐ Managed/protected well
- ☐ Undeveloped/protected well (traditional)
- ☐ Surface water (rivers, dams, lakes, ponds, irrigation channels)
- ☐ Others

Where do the members of your household get their main relief?

- ○ Your toilet
- ○ Outside latrine (school, market, etc.)
- ○ Neighbor's toilet
- ○ Common latrine
- ○ In the wild
- ○ Other locations
- ○ Don't know / refuse to answer

What type of latrine does your household use?

- ◯ EcoSan latrine
- ◯ Latrine Sanplat
- ◯ VIP latrine
- ◯ Septic tank
- ◯ TCM latrine
- ◯ Traditional toilet
- ◯ In the wild

When do members of your household wash their hands?

- ☐ After using the toilet
- ☐ Before preparing food
- ☐ After changing an infant's diaper, wiping a child's bottom or helping a child use the toilet
- ☐ Before eating
- ☐ After eating
- ☐ Before feeding the children
- ☐ After touching animals
- ☐ Before handling food
- ☐ Before praying
- ☐ Other

Does your household have a running-water hand-washing system?

- ◯ Yes
- ◯ No

Describe this hand washing system

____

Is there water in this device?

- ◯ Yes
- ◯ No

Is soap available in the hand-washing system?

- ◯ Yes
- ◯ No

What type of soap does the household usually use for hand washing?

- ◯ Ball soap
- ◯ Liquid soap
- ◯ Soapy water
- ◯ Other

Cereal consumption at home yesterday

Sorghum, sorghum cream, sorghum couscous, sorghum tô; millet (small millet), millet/degue/thiamguiri cream, millet couscous, millet plate, millet plate (flour/breakage), maize (broken or flour), sweet grilled corn, cornmeal, fonio, fonio plate, rice, pasta (macaroni, etc.), wheat, bread, millet/wheat pâté, wheat flour, millet/rice patties, corn/millet/rice porridge, millet/corn/rice fritters, fortified flour

- ◯ Yes
- ◯ No

Tuber and root consumption at home yesterday

White sweet potato, potato, manioc, taro, plantain (aloco), yam, roast root, water lily root, turnip root, white sweet potato-based tofu.

- ◯ Yes
- ◯ No

Consumption of legumes at home yesterday

Beans (cowpea), potato/woandzou peas, sweet peas, chickpeas, lentils, soybeans, other

- ◯ Yes
- ◯ No

Eating nuts and seeds at home yesterday

Water lily seeds, peanuts (in paste or plain, oil cakes), sesame, wild walnuts, boscia nuts, cashew nuts, wild walnuts, date or soumbala, bikalga (fermented sorrel seeds).

- ◯ Yes
- ◯ No

Milk and dairy products consumption at home yesterday

Fresh milk, powdered milk, condensed milk (sweetened or not), natural curd, yoghurt, cheeses...

- ◯ Yes
- ◯ No

Consumption of offal at home yesterday

Liver, kidneys, heart, lungs, or any other offal (veal, sheep, goat, poultry), viscera (viscera soup)...

- ◯ Yes
- ◯ No

Meat and meat products consumption at home yesterday

Beef, sheep, goat, rabbit, chicken, guinea fowl, birds, duck bush meat (gazelle, monitor fish, turtle, wild rats, agoutis, squirrels, partridge, snake, mouse, deer, etc.), frog, crapeau...

- ◯ Yes
- ◯ No

Fish and seafood consumption at home yesterday

Fresh fish, smoked fish, salted fish, canned fish (sardines, tuna, etc.), shrimps, snails, crab, etc.

- ◯ Yes
- ◯ No

Eating eggs at home yesterday

Chicken, guinea fowl, duck, quail, goose eggs...

- ◯ Yes
- ◯ No

Consumption of dark green leafy vegetables at home yesterday

Baobab leaves, dark green shallot leaves, onion leaves, koumba leaf, spinach, dark green salad, lélé leaves, fakoye leaves, all dark green wild leaves

- ◯ Yes
- ◯ No

Eating vitamin A-rich vegetables, roots and tubers at home yesterday

Carrot, red pepper, orange-fleshed sweet potato, orange or dark yellow flesh squash (orange flesh squash plate), orange flesh sweet potato plate, potato leaves, cassava leaves, sorrel leaves (dah), squash leaves, bean leaves, moringa leaves

- ◯ Yes
- ◯ No

Eating vitamin A-rich fruit at home yesterday

Mango, papaya, pumpkin, orange-fleshed melon, néré/powdered néré

- ◯ Yes
- ◯ No

Consumption of other vegetables at school yesterday

Fresh tomatoes, fresh or dried okra, eggplants, local eggplants (jaxatus or yalo), zucchinis, cucumbers, cabbages, onions, fresh shallots, green peppers, green beans, lettuce (salad), spinach...

- ◯ Yes
- ◯ No

Consumption of other fruit at school yesterday

Banana, guava, watermelon, orange, lemon, dates, jujube, wild fruits ("grape"/bembe, monkey bread/baobab fruit), wild "dates" (Aduwa), shea pulp, liana fruit, roasted fruit flesh, pineapple, avocado...) Fresh fruit juices (pressed fruit)...

- ◯ Yes
- ◯ No

Consumption of red palm oil at home yesterday

Red palm oil, foods prepared with red palm nuts or red palm nut pulp...

- ◯ Yes
- ◯ No

Insect consumption at home yesterday

Insects, caterpillars, insect eggs, locusts/flycatchers, mayflies (yiou)...

- ◯ Yes
- ◯ No

Oil and fat consumption at home yesterday

Vegetable oil (peanut, sesame, coconut, etc.), shea butter/oil, cow's butter (sirimè), vegetable fats/margarine, mayonnaise, animal fats. - for sauces, seasonings, frying...), shea butter/oil, cow's butter (sirimè), vegetable fats/margarine, mayonnaise, animal fats...

- ◯ Yes
- ◯ No

Sugar consumption and sweet products at home yesterday

Powder or lump sugar (in tea, coffee, porridge, etc.), sweet drinks (soft drinks/sugar, bissap, ginger juice, sweet tamarind leaf or fruit juice, lemon grass), honey, jam, sweets, sweet doughnuts, sweet cakes, sweet cookies, tea, coffee, chikoré, kinkeliba, unsweetened tamarind leaf or fruit juice.

- ◯ Yes
- ◯ No

Consumption of condiments and spices at home yesterday

Chilli, pepper, vinegar, garlic, spice sachet, salt, Maggi cube, white Maggi, bay leaf, concentrated tomato, sorrel-based condiment (seed/datou, leaves or pulp), onion-based condiment or dried/processed onion leaves or soy-based condiment, small amount of fish powder, okra powder, dried baobab leaf, bell pepper, soumbala.

- ◯ Yes
- ◯ No
